# Soil Bacterial Communities Exhibit Strong Biogeographic Patterns at Fine Taxonomic Resolution

**DOI:** 10.1128/mSystems.00540-20

**Published:** 2020-07-21

**Authors:** Sean K. Bay, Melodie A. McGeoch, Osnat Gillor, Nimrod Wieler, David J. Palmer, David J. Baker, Steven L. Chown, Chris Greening

**Affiliations:** a School of Biological Sciences, Monash University, Clayton, VIC, Australia; b Department of Microbiology, Biomedicine Discovery Institute, Clayton, VIC, Australia; c Zuckerberg Institute for Water Research, Blaustein Institutes for Desert Research, Ben Gurion University of the Negev, Sde Boker, Israel; University of California San Diego

**Keywords:** biogeography, desert, soil bacteria, turnover, zeta diversity

## Abstract

Bacteria have been inferred to exhibit relatively weak biogeographic patterns. To what extent such findings reflect true biological phenomena or methodological artifacts remains unclear. Here, we addressed this question by analyzing the turnover of soil bacterial communities from three data sets. We applied three methodological innovations: (i) design of a hierarchical sampling scheme to disentangle environmental from spatial factors driving turnover; (ii) resolution of 16S rRNA gene amplicon sequence variants to enable higher-resolution community profiling; and (iii) application of the new metric zeta diversity to analyze multisite turnover and drivers. At fine taxonomic resolution, rapid compositional turnover was observed across multiple spatial scales. Turnover was overwhelmingly driven by deterministic processes and influenced by the rare biosphere. The communities also exhibited strong distance decay patterns and taxon-area relationships, with *z* values within the interquartile range reported for macroorganisms. These biogeographical patterns were weakened upon applying two standard approaches to process community sequencing data: clustering sequences at 97% identity threshold and/or filtering the rare biosphere (sequences lower than 0.05% relative abundance). Comparable findings were made across local, regional, and global data sets and when using shotgun metagenomic markers. Altogether, these findings suggest that bacteria exhibit strong biogeographic patterns, but these signals can be obscured by methodological limitations. We advocate various innovations, including using zeta diversity, to advance the study of microbial biogeography.

**IMPORTANCE** It is commonly thought that bacterial distributions show lower spatial variation than for multicellular organisms. In this article, we present evidence that these inferences are artifacts caused by methodological limitations. Through leveraging innovations in sampling design, sequence processing, and diversity analysis, we provide multifaceted evidence that bacterial communities in fact exhibit strong distribution patterns. This is driven by selection due to factors such as local soil characteristics. Altogether, these findings suggest that the processes underpinning diversity patterns are more unified across all domains of life than previously thought, which has broad implications for the understanding and management of soil biodiversity.

## Abstract (precis)

It is commonly thought that bacterial distributions show lower spatial variation than for multicellular organisms. In this article, we present evidence that these inferences are artifacts caused by methodological limitations. Through leveraging innovations in sampling design, sequence processing, and diversity analysis, we provide multifaceted evidence that bacterial communities in fact exhibit strong distribution patterns. This is driven by selection due to factors such as local soil characteristics. Altogether, these findings suggest that the processes underpinning diversity patterns are more unified across all domains of life than previously thought, which has broad implications for the understanding and management of soil biodiversity.

## INTRODUCTION

A central goal of microbial ecology is to link microbial distribution patterns to underlying ecological processes. Developing such links is important both for fundamental science and applied outcomes, for example to make accurate global biodiversity assessments and prioritize management goals in the face of both local and global change (1, 2). However, achieving this critically depends on our abilities to adequately characterize biodiversity at the first stage, with various methodological and theoretical challenges limiting our understanding of microbial distribution patterns and their underlying ecological drivers.

Several principles have nevertheless become established in soil microbial ecology through cultivation-independent studies over the last two decades. First, it is appreciated that most soils harbor rich and abundant bacterial communities (3–6). In most soils, a small number of taxa are abundant and prevalent, while the remaining taxa have low abundance and frequency (the “rare” biosphere) (7, 8). In common with macroorganisms (9), four key ecological processes control microbial assembly across space and time: environmental selection, diversification, dispersal, and drift (10–12). While much work has emphasized the role of deterministic environmental selection in driving bacterial niche differentiation, especially edaphic factors such as pH (13–17), some studies have also inferred stochastic patterns of community structure, for example due to dispersal limitation or historical diversification (17–21). The relative strength of these factors can vary across time, for example with dispersal controlling recruitment and selection affecting retention during initial stages of primary succession (17, 22–24). As is also the case in the field of macroecology, the relative importance of deterministic and stochastic processes in shaping contemporary distributions of microorganisms continues to be debated and there is a large body of often divergent literature in this area. In this regard, a major methodological challenge is to perform sampling and analysis that sufficiently disentangles the autocorrelation between environmental and spatial factors in soil ecosystems (12, 25–27).

Also controversial is the extent to which microbial communities vary across space. Soil bacteria are generally thought to exhibit weaker biogeographic patterns than macroorganisms (15, 28). Most empirical studies have reported low exponents for taxa-area relationships (13, 15, 29–31) and low regression coefficients in distance decay curves (13, 19, 28, 32, 33), though exceptions have been reported (34–37). Several hypotheses have been put forward to explain these observations (38, 39). Primarily, bacteria are thought to be able to maintain wide geographic ranges in the face of environmental variation by entering dormant states (39, 40), leading to limited geographic turnover and shallow taxon-area curves (15, 28, 41). However, methodological artifacts may also account for some observations of weak spatial differences (28). Microbial biogeographic patterns are known to be sensitive to various factors, including spatial scale (42, 43), sampling effort (28, 44–46), and taxonomic resolution (15, 28, 44, 47–49). Communities are inherently prone to being undersampled, whether through insufficient sampling effort, low sequencing depth, or rarefying data (50, 51). In addition, the processing of 16S rRNA gene amplicon sequencing data typically used to profile communities can reduce data set resolution; reads are usually clustered into operational taxonomic units (OTUs) based on an arbitrary identity threshold (usually 97%), and the rare biosphere is regularly removed (52, 53). Compounding these issues, the pairwise analyses generally used to quantify community turnover inadequately partition variation from all community members: incidence-based measures are highly sensitive to the rare biosphere, and abundance-based measures focus on the common few (54, 55).

In this study, we employed three methodological innovations to address these common limitations of microbial biogeographic surveys and reassess patterns of bacterial community turnover. First, we adopted a hierarchical sampling scheme commonly used in macroecological surveys (56, 57); this enabled us to detect changes in community structure across multiple spatial scales and, in light of controversies in the literature, better distinguish the contributions of environmental and spatial drivers to community assembly processes (27). Second, we profiled community composition using high-resolution 16S rRNA gene amplicon sequence variants (ASVs), leveraging a new generation of sequence processing tools (58–60). We compared the effects of the commonly used approaches of filtering and clustering sequences on calculated community turnover; this is important given that clustering sequences reduces taxonomic resolution and thus may increase the overall similarity of the community, thereby weakening biogeographic patterns (35, 49). Finally, we used the multisite diversity metric zeta diversity to analyze spatial community turnover and predict the strength of underlying deterministic and stochastic drivers (55). Unlike the commonly used beta diversity that is calculated from pairwise comparisons, zeta diversity describes the number of taxa shared across multiple sites. As a result, this parameter can discriminate diversity patterns across the spectrum of common, intermediate, and rare taxa (55, 61–63) and infer deterministic and stochastic drivers of community assembly. On this basis, we provide evidence that at the level of exact sequences, bacterial biogeographic patterns are exceptionally stronger than previously reported.

## RESULTS

### Most community members have a low to moderate occupancy across soil transects.

We analyzed 96 topsoil samples along two perpendicular transects (see Fig. S1a in the supplemental material): a 160-km latitudinal transect (north/south) spanning four climatic zones (subhumid, semiarid, arid, and hyperarid; 69 samples) and a 20-km longitudinal transect (east/west) in the arid zone (27 samples). Within each transect, samples were collected according to a hierarchical design (2 sites per zone × 3 plots per site × 3 samples per plot) (Fig. S1b). This sampling scheme was designed to enable the analysis of microbial community turnover at multiple spatial scales, capture a wide spectrum of distance classes (Fig. S1c), and discriminate underlying spatial and environmental drivers.

10.1128/mSystems.00540-20.1 FIG S1 Details of study site and sampling design. (a) Map of study site ranging from the Judea Hills in the north to the Negev region in the south of Israel. The left panel shows the location of the study site (blue enclosed circle) within a world map shaded by the aridity index. The right panel shows the locations of the sampling sites. Samples were collected across a 160-km latitudinal (north/south) transect and a 20-km longitudinal (east/west) transect. The latitudinal transect spanned a steep aridity gradient, and samples were collected from four climatic zones (subhumid, semiarid, arid, and hyperarid). The photographs on the right were taken during the sampling campaign, showing vegetation cover and geographic features of each climatic zone. For the longitudinal transect, all samples were collected in the arid zone. (b) Details of the hierarchically nested sampling design. Samples were collected from four climatic zones along the aridity gradient (subhumid, semiarid, arid, and hyperarid). There were three hierarchies of spatial sampling within each climatic zone: (i) two sites were sampled at each zone (site 1 and site 2); (ii) three different plots were sampled at each site (plot A, plot B, and plot C); and (iii) three random soil samples were collected from each plot (sample 1, sample 2, and sample 3). (c) Frequency distribution of sampling distances between the 96 sampling sites. Pairwise distances are showed at a resolution of 10-km intervals. This analysis confirms that most distance classes were represented (unrepresented bins, 110 km and 140 km) across the study site and that all represented distance classes were associated with >100 site pairs (minimum = zero, 1st quartile = 24.6 km, median = 42.5 km, mean = 50.5 km, 3rd quartile = 68.8 km, maximum = 155.5 km). Download FIG S1, TIF file, 2.7 MB. Copyright © 2020 Bay et al. 2020 Bay et al. This content is distributed under the terms of the Creative Commons Attribution 4.0 International license.

**FIG 1:**
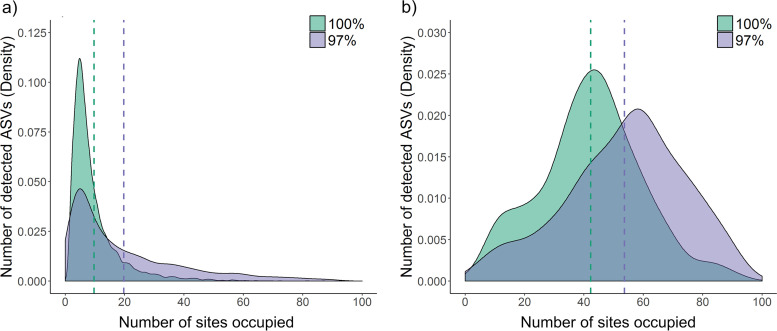
Occupancy frequency distribution of amplicon sequence variants (ASVs) at different taxonomic resolutions. The Kernel-smoothed density plot shows the number of sites that each taxon (ASVs) was detected in across the data set. (a) Effect of clustering taxa at either 100% or 97% identity threshold. (b) Effect of either including or removing taxa with lower than 0.05% relative abundance. The vertical dashed lines show distribution means.

The bacterial and archaeal communities in each sample were profiled using both new and standard approaches for processing 16S rRNA gene amplicon sequencing data. Rarefaction curves (Fig. S2a to c) and richness estimators (Fig. S2d) confirmed that sequencing and sampling efforts sufficiently captured the diversity of taxa within and across samples. A high-resolution community profile was generated by processing reads using the deblur pipeline (59) to resolve 16S rRNA gene amplicon sequence variants (ASVs) at the single-nucleotide level (singletons removed) (see Data Set S1, tab 1, in the supplemental material). Most sequences were from the nine dominant soil phyla (7), especially *Actinobacteriota*, *Chloroflexota*, and *Proteobacteria*, as well as putatively ammonia-oxidizing archaea (Fig. S3). The occupancy frequency distribution (64) of the 11,335 taxa (ASVs) detected was positively skewed; ∼67% of 7,602 taxa were detected in fewer than 10% of samples (Fig. 1a).

10.1128/mSystems.00540-20.2 FIG S2 Alpha diversity of individual samples and wider transects. (a and b) Sample-based alpha rarefaction curves. The curves show number of taxa (ASVs) detected relative to the number of sequencing reads at identity thresholds of (a) 100% and (b) 97%. (c) Taxa accumulation curve showing number of taxa (ASVs) detected across all sites. Points show cumulative sample richness, and error bars show the estimated standard deviation at identity thresholds of 100% and 97%. (d) Observed and estimated community richness. The site level observed and estimated richness of taxa (ASVs) is shown across four climatic zones, subhumid (SH), semiarid (SA), arid (AR), hyperarid (HA), and three sites along the longitudinal transect (LO 1 to 3). Estimated richness was calculated using the Chao1 method and abundance coverage estimate (ACE). Box plots show medians, upper quartiles, and lower quartiles at two taxonomic resolutions. Download FIG S2, TIF file, 1.4 MB. Copyright © 2020 Bay et al. 2020 Bay et al. This content is distributed under the terms of the Creative Commons Attribution 4.0 International license.

10.1128/mSystems.00540-20.3 FIG S3 Phylum-level community structure. Bars represent relative abundance of bacteria and archaea detected by amplicon sequencing of 16S rRNA genes. Sample identity are given along with corresponding climatic zone and sampling transect. Sequences were assigned based on Genome Taxonomy Database (GTDB) taxonomy. Download FIG S3, TIF file, 3.2 MB. Copyright © 2020 Bay et al. 2020 Bay et al. This content is distributed under the terms of the Creative Commons Attribution 4.0 International license.

10.1128/mSystems.00540-20.9 DATA SET S1 Summary of community composition and physicochemical characteristics of the soil samples. Tab 1: Sequences and read counts of each amplicon sequence variant shown per sample denoised at 100% sequence identity. Tab 2: Sequences and read counts of each amplicon sequence variant shown per sample clustered at 97% sequence identity. Tab 3: Geographic and chemical characteristics of soils collected along the latitudinal and longitudinal transects. Download Data Set S1, XLSX file, 4.5 MB. Copyright © 2020 Bay et al. 2020 Bay et al. This content is distributed under the terms of the Creative Commons Attribution 4.0 International license.

We then compared the effects of applying two standard approaches used to process sequencing data into OTUs: (i) clustering, i.e., combining sequences with an identity threshold of 97%, and (ii) filtering, i.e., removing sequences with lower than 0.05% relative abundance (Data Set S1, tab 2). There was a sharp decrease in the number of taxa retained (2,943 clustered, 222 filtered, 403 clustered then filtered). Though clustering inevitably reduced richness, as well as the frequency of intermediate taxa, it did not affect the skew of the occupancy frequency distribution (Fig. 1a). However, when less abundant taxa were filtered from the data sets, occupancy frequency shifted from a positive skew to a modal distribution (Fig. 1b). These findings suggest that the prevalence of most community members is low to moderate; standard clustering and filtering approaches not only affect the “rare” biosphere, but a large percentage of community members with moderate range sizes. In turn, changing occupancy properties may underestimate ecological heterogeneity and markedly bias biogeographic interpretations.

### Deterministic factors drive differences in community composition between soil samples.

**FIG 2:**
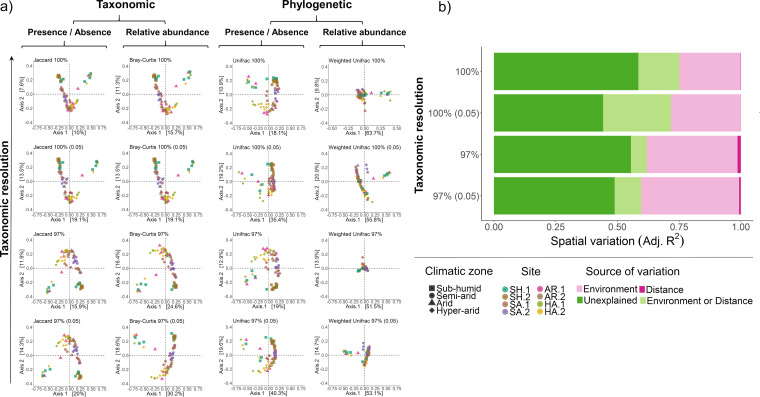
Patterns and drivers of beta diversity in the latitudinal transect. (a) Multidimensional scaling visualizing taxonomic and phylogenetic pairwise incidence and abundance dissimilarity of microbial communities. The axes show the explained variation of taxa (ASVs) between samples using four different dissimilarity metrics: Jaccard (taxonomic incidence-based), Bray-Curtis (taxonomic abundance-based), unweighted Unifrac (phylogenetic incidence-based), and weighted Unifrac (phylogenetic abundance-based) dissimilarity metrics. The PCoA ordination is compared at four different taxonomic resolutions (taxa clustered at 100% or 97% identity; taxa with <0.05% relative abundance included or removed). (b) Variation partitioning analysis delineating the relative contributions of environmental and spatial sources of variation on microbial community structure. The analysis shows the proportion of variation in microbial incidence between sample pairs as explained by environmental, spatial, overlapping, and unexplained sources of variation. These analyses were performed using data from each plot in the latitudinal transect. Results are compared at four different taxonomic resolutions (taxa clustered at 100% or 97% identity; taxa with <0.05% relative abundance included or removed). Data Set S2, tab 3, in the supplemental material summarizes the environmental variables that best explain the variation along the transect.

We subsequently used pairwise metrics (beta diversity) to analyze community composition between samples. We detected significant differences in microbial richness down to the site level (Data Set S1, tab 1) and community structure down to the plot level (Data Set S2, tab 2). The extent of compositional differences observed between sites depended on both the community property used (incidence versus abundance, taxonomic versus phylogenetic) and the taxonomic resolution of the data set. Principal-coordinate analysis (PCoA) ordinations showed prominent “V” patterns (Fig. 2a); this pattern, also known as the horseshoe effect, has been shown to indicate the presence of niche differentiation along environmental gradients (65). In line with the high environmental heterogeneity along the latitudinal transect, differentiation was more pronounced for the latitudinal transect than for the longitudinal transect (Fig. S4a).

10.1128/mSystems.00540-20.4 FIG S4 Patterns and drivers of pairwise and multisite turnover in the longitudinal transect. (a) Multidimensional scaling visualizing taxonomic and phylogenetic pairwise incidence and abundance dissimilarity of microbial communities. The axes show the explained variation of taxa (ASVs) between samples using four different dissimilarity metrics: Jaccard (taxonomic incidence-based), Bray-Curtis (taxonomic abundance-based), unweighted Unifrac (phylogenetic incidence-based), and weighted Unifrac (phylogenetic abundance-based) dissimilarity metrics. The PCoA ordination is compared at four different taxonomic resolutions (taxa clustered at 100% or 97% identity; taxa with <0.05% relative abundance included or removed). (b) Variation partitioning analysis delineating the relative contributions of environmental and spatial sources of variation on microbial community structure. The analysis shows the proportion of variation in microbial incidence between sample pairs as explained by environmental, spatial, overlapping, and unexplained sources of variation. These analyses were performed using data from each plot in the latitudinal transect. Results are compared at four different taxonomic resolutions (taxa clustered at 100% or 97% identity; taxa with <0.05% relative abundance included or removed). Data Set S2, tab 4, summarizes the environmental variables that best explain the variation along the transect. (c) Distance decay relationship showing community turnover with increasing geographic distance based on pairwise comparisons. (d) Differences in the slope (coefficient) of distance decay when moving from pairwise comparisons to higher orders of zeta (>2) using the average distance between higher orders of zeta. (e) Taxa-area relationship showing increase in richness with area sampled. Download FIG S4, TIF file, 1.6 MB. Copyright © 2020 Bay et al. 2020 Bay et al. This content is distributed under the terms of the Creative Commons Attribution 4.0 International license.

10.1128/mSystems.00540-20.10 DATA SET S2 Summary of statistical analyses performed for study. Tab 1: One-way ANOVA showing differences of observed and estimated alpha diversity. Results are shown between sites at two taxonomic resolutions. Tab 2: One-way ANOVA results showing differences in community structure. Results are shown at zone, site, plot, and sample scales at four taxonomic resolutions using likelihood ratio test (LRT). Tab 3: Subset of independent variables that best explain community variation along the latitudinal transect. Results show total *R*^2^ obtained from a forward selection in a multivariate linear model. The results are shown for models of each of the four taxonomic resolutions. Tab 4: Subset of independent variables that best explain community variation along the longitudinal transect. Results show total *R*^2^ obtained from a forward selection in a multivariate linear model. The results are shown for models of each of the four taxonomic resolutions. Tab 5: Summary statistics of general linear models of zeta diversity distance decay. Results are shown for the latitudinal and longitudinal transect for each of the four taxonomic resolutions. Tab 6: Comparative analysis of values for exponent *z* of the species-area relationship previously reported for both eukaryotic and prokaryotic communities in comparison to this study. Studies are ranked by *z* value. Download Data Set S2, XLSX file, 0.02 MB. Copyright © 2020 Bay et al. 2020 Bay et al. This content is distributed under the terms of the Creative Commons Attribution 4.0 International license.

A variation partitioning analysis was used to delineate the measured environmental and spatial predictor variables that best explain pairwise community structure. Across the latitudinal gradient, 45% of the community variation of the high-resolution data set was explained by measured edaphic factors (Fig. 2b; Data Set S1, tab 3) with pH, C/N ratio, aridity, and salinity explaining the greatest amount of variation (Data Set S2, tab 3). These results broadly reflect other studies in the Negev region and along aridity gradients globally (66–68). Less variation was explained for the more homogeneous longitudinal transect (35%) (Fig. S4; Data Set S2, tab 4). Altogether, these results suggest environmental effects predominate over distance effects in driving community composition.

In common with other biogeographic studies (13, 69, 70), a large proportion of variation was unexplained by the measured variables. A combination of factors could contribute to this unexplained variation, including deterministic processes driven by unmeasured abiotic and biotic factors, as well as neutral ecological drift and potentially sampling effects. In both the PCoA and variation partitioning analyses, less variation in community composition could be explained and partitioned for the high-resolution data set compared to filtered ones (Fig. 2 and Fig. S4). The rank importance and weight of environmental predictors also shifted depending on taxonomic resolution for both transects (Data Set S2, tabs 3 and 4). In support of recent findings (71), these results suggest that different environmental drivers structure common and rare microbial taxa.

### Soil microbial communities exhibit rapid deterministically driven multisite turnover.

We also analyzed spatial turnover in the community using the recently developed metric zeta diversity. As depicted in the infographic in Fig. S5, zeta diversity describes the number of taxa shared by multiple combinations of sites; whereas beta diversity (which it encompasses) is predisposed to detect turnover of rare taxa, zeta diversity discriminates patterns and drivers of turnover across the spectrum of common, intermediate, and rare taxa (55, 61).

10.1128/mSystems.00540-20.5 FIG S5 Infographic describing zeta diversity. This shows how zeta diversity, unlike beta diversity, can provide information on the contribution of rare, intermediate, and common taxa to community turnover. A complementary infographic showing the effects of clustering and filtering on zeta decline and zeta distance decay, within the context of this study, is shown in Fig. 5. Download FIG S5, TIF file, 1.0 MB. Copyright © 2020 Bay et al. 2020 Bay et al. This content is distributed under the terms of the Creative Commons Attribution 4.0 International license.

**FIG 3:**
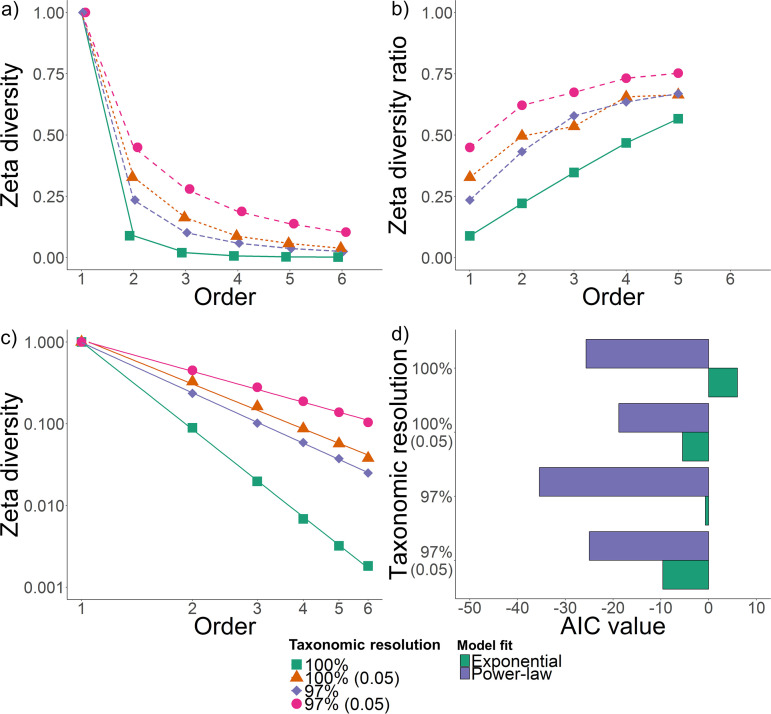
Multisite community turnover and assembly processes along the latitudinal transect at different taxonomic resolutions. (a) Zeta decline showing how the number of shared taxa (ASVs) decline with the addition of sites to the comparison (Order). (b) Zeta diversity ratio showing rate of taxon retention. This demonstrates the probability of retaining common over rare taxa at any particular order with the addition of an extra site. (c) Zeta decline followed a power law form in all cases, which is associated with deterministic processes driving community turnover. (d) Statistical support for the power law form, relative to an exponential form, varies depending on the taxonomic resolution.

For the high-resolution data set, zeta diversity rapidly declined toward zero within four orders in the latitudinal transect (ζ_4_ = 0.0068) (Fig. 3a). This means that the average number of taxa shared across any four plots was 0.68% of 10,826, indicating very rapid turnover. Similar patterns were observed across both transects and within each climatic zone; somewhat lower turnover was observed along the longitudinal transect (ζ_4_ = 0.010) (Fig. S6). Reducing taxonomic resolution markedly slowed compositional turnover (Fig. 3a); for the clustered and filtered data set, up to 30% of the community were shared across any four plots (ζ_4_ = 0.18 and 0.30 for the latitudinal and longitudinal transects, respectively). Such findings reflect that, given that common, intermediate, and rare community members show different distribution patterns, lowering taxonomic resolution distorts detection of microbial turnover and underlying drivers.

10.1128/mSystems.00540-20.6 FIG S6 Normalized zeta diversity decline showing the compositional turnover in taxa (ASVs) across sites at different taxonomic resolutions. Zeta decline (a, d, g, and j) quantifies how the number of shared taxa declines with increasing orders of zeta (number of sites included in the calculation of zeta). The functional decline frequently follows either an exponential (equal probability of taxa occurrence across sites) or a power law form (unequal probability of taxa occurrence across sites), which reflects turnover being driven largely by either stochastic (exponential) and deterministic (power law) community assembly processes. In all cases, the decline followed a power law form (b, e, h, and k), though the goodness of fit varied depending on taxonomic resolution. The taxa retention rate using zeta ratio (c, f, i, and l) quantifies the probability of retaining common over rare taxa at any particular order with the addition of an extra site. (i) Statistical support for power law and exponential model fits of zeta diversity decline. The bar plots show AIC values of power law and exponential general linear model fits for the two transects (latitudinal and longitudinal) and four climatic zones (from subhumid to hyperarid). Results are compared at four different taxonomic resolutions (taxa clustered at 100% or 97% identity; taxa with <0.05% relative abundance included or removed). Download FIG S6, TIF file, 1.5 MB. Copyright © 2020 Bay et al. 2020 Bay et al. This content is distributed under the terms of the Creative Commons Attribution 4.0 International license.

Derivations show that zeta decline most often follows either a power law or an exponential form, which are, respectively, associated with either deterministic or stochastic community assembly processes (55). Zeta decline much better fitted a power law form for both transects and within each climatic zone (Fig. 3c and Fig. S6). This therefore suggests that deterministic processes drive turnover and rejects the null hypothesis that microbial communities are randomly distributed. While power law support was overwhelming for the high-resolution data set, there was some support for exponential models in the low-resolution data sets; filtering microbial data sets, by obscuring biogeographic structure, may therefore cause false signals of stochastic assembly processes (Fig. S6).

### Soil microbial communities exhibit strong distance decay and taxon-area relationships.

**FIG 4:**
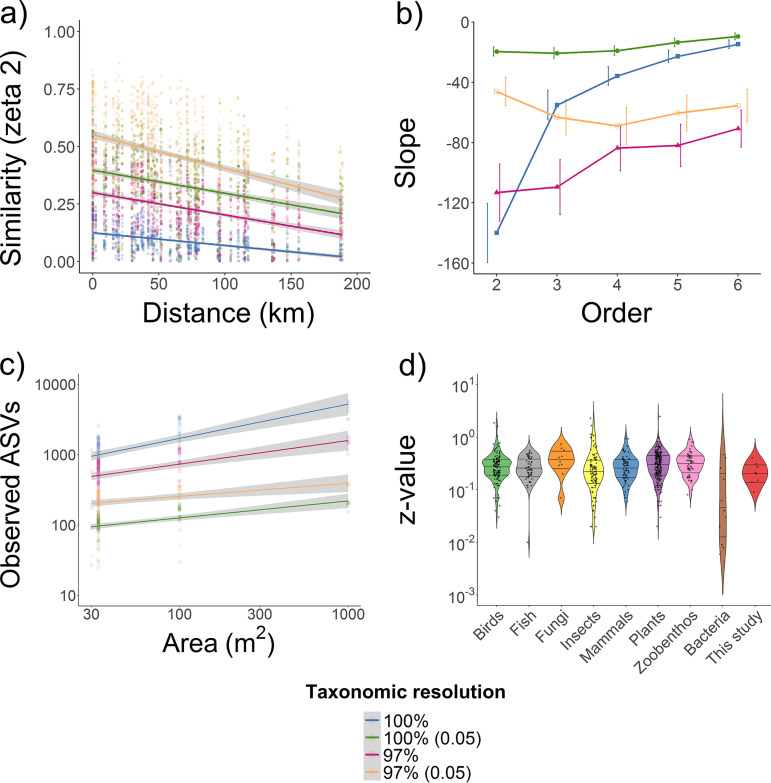
Distance decay in community similarity and the taxa-area relationship at different taxonomic resolutions. (a) Zeta distance decay relationship showing community turnover with increasing geographic distance based on pairwise comparisons (ζ_2_) of sites along the latitudinal transect. (b) Differences in the slope (coefficient) of distance decay between pairwise and higher orders of zeta (>2) using the average distance between sites. (c) Taxa-area relationships of the increase in richness with area sampled along the latitudinal transect. This shows effects of taxonomic resolution for understanding how compositional heterogeneity scales with distance. (d) Violin plots showing the density distribution and interquartile range of the exponent *z* of the taxa-area slope reported here with those from other studies for bacteria and eukaryotes (Data Set S2, tab 6). Results are compared at four different taxonomic resolutions, whereby (i) taxa were clustered at either 100% or 97% identity threshold and (ii) taxa with lower than 0.05% relative abundance were either included or removed.

We subsequently measured distance decay using a combination of pairwise (beta decay) and multisite (zeta decay) metrics. Based on pairwise comparisons, a strong decay of shared taxa was also detected across transects (*P* < 0.0001) (Fig. 4a and Fig. S4c; Data Set S2, tab 5). Lowering taxonomic resolution caused a large increase in community similarity, a steeper distance decay coefficient, and a lower rate of community turnover overall; across the 160-km latitudinal transect, there was a 82% reduction in community similarity for the high-resolution data set compared to 50% to 60% reductions for the clustered and/or filtered data sets. Given the concordant support for deterministic drivers, based on the zeta diversity (Fig. 3), variation partitioning analysis (Fig. 2b), and PCoA analysis (Fig. 2a), these decay patterns likely reflect environmental filtering rather than dispersal limitation.

To quantify how distance decay compares between rare, intermediate, and common taxa, distance decay was calculated for up to six zeta orders by using the mean distance between pairs for up to six plots. For both transects at high resolution, the gradient of the distance decay curve rapidly and significantly decreased with increasing zeta order (Fig. 4b and Fig. S4d). This provides additional evidence that these microbial communities are highly structured and that turnover is driven by loss of rare to intermediate members. In contrast, there were no significant changes in distance decay rates with zeta order for the less-resolved data sets, further demonstrating that clustering and/or filtering obscures biogeographic patterns.

Given these outcomes, we revisited the controversial taxa-area relationship for bacterial communities (15, 44) using these data sets. This universal relationship in ecology describes the increase in taxon richness with area sampled, i.e., *S* = *cA^z^* (where *S* is number of species, *A* is area sampled, and *c* and *z* are fitted constants), and its exponent *z* is a normalized measure of turnover rates that can be compared between organismal groups (15). A strong taxa-area relationship was also observed for both transects (*P* < 0.001) (Fig. 4c; Fig. S4e). The *z* exponents were 0.39 (latitudinal transect) and 0.40 (longitudinal transect) for the original high-resolution data sets, and the exponents decreased to 0.13 and 0.09 in the clustered then filtered data sets (Data Set S2, tab 6). Such *z* exponents greatly exceed those reported for bacterial communities in most previous studies (median, 0.04), but are congruent with four studies (29, 34, 35, 37), two of which also performed hierarchical sampling. These exponents are of the same order of magnitude as those previously reported for animal and plant data sets (median, 0.27) (Fig. 4d; Data Set S2, tab 6), indicating that biogeographic patterns of bacteria and macroorganisms may not profoundly differ. However, more broad and detailed side-by-side sampling is required to compare scaling relationships between bacteria and macroorganisms.

### Similar biogeographic patterns are observed using metagenomic sequences and global data sets.

This study relies on 16S rRNA gene amplicon sequencing to profile the soil microbial communities. This approach remains standard practice for biogeographic studies, given that the alternative of metagenomic profiling requires much higher sequencing depths and yields either less information-rich short reads or more error-prone long reads (72). However, limitations of 16S rRNA gene sequencing include potential for amplification and sequencing errors, biases in the primer sets, and genome variability in 16S rRNA gene copy number (73). While it is possible that the data set includes some spurious sequences introduced through this approach, these are unlikely to account for the surprising observations made here. First, a range of accuracy measures suggest deblur efficiently denoises sequencing data and that a 100% identity threshold resolved using the deblur denoising pipeline is optimal for community profiling with the V4 region (58, 59). Second, similar but weaker patterns of rapid deterministically driven community turnover were observed for the clustered (but not filtered) data sets, in which most spurious sequences should be removed (Fig. 2 and 3).

To test the reproducibility of our findings, we performed short-read metagenomic sequencing of a subset of 12 samples across the latitudinal transect and analyzed a single-copy ribosomal marker gene, *rplP*. Similar to the 16S rRNA gene amplicon data, samples showed a high estimated richness, comparable taxonomic composition, and rapid community turnover (Fig. S7). Zeta decline approached zero after three orders (ζ_3_ = 0.06) using a rarefied data set (Fig. S7c). In combination, this suggests that 16S rRNA gene ASVs are sufficient to estimate community turnover, whereas standard methods of clustering and filtering data obscure biogeographic patterns and inflate signals of taxon commonness.

10.1128/mSystems.00540-20.7 FIG S7 Comparison of community diversity, composition, and turnover based on amplicon and metagenomic sequencing. The bacterial and archaeal community composition of 12 sites across the latitudinal gradient was determined by either amplicon sequencing of the multicopy 16S rRNA gene V4 region or shotgun metagenomic sequencing of the single-copy ribosomal protein gene *rplP*. (a) Observed and estimated richness. Estimated richness was calculated using the Chao1 method and abundance coverage estimate (ACE). Box plots show medians, upper quartiles, and lower quartiles at two taxonomic resolutions. (b) Phylum-level community composition across the sites based on the 16S rRNA gene and *rplP* gene. Taxonomic assignment is based on the Genome Taxonomy Database (GTDB). (c) Normalized zeta diversity decline showing compositional turnover in taxa from 16S rRNA gene ASVs at two identity thresholds (100% and 97%) and the single-copy ribosomal marker gene *rplP* clustered at 97%. Download FIG S7, TIF file, 2.0 MB. Copyright © 2020 Bay et al. 2020 Bay et al. This content is distributed under the terms of the Creative Commons Attribution 4.0 International license.

Having detected these patterns at local and regional scales, we analyzed whether similar patterns were observable at the continental scale. To do so, we analyzed previously published 16S rRNA gene profiles of 237 soil samples collected from six continents (7). As with our original data set, we processed the 16S rRNA amplicon sequencing data into ASVs and analyzed the effects of clustering and/or filtering. The occupancy frequency distribution of the taxa showed a skew similar to that of the Israel data set (Fig. S8). Concordant with our previous observations, zeta diversity rapidly declined across the first few orders and followed a power law relationship with strong model support (Fig. S8). Clustering and filtering altered the occupancy frequency distribution, resulting in ∼10% to 30% of taxa being retained at six zeta orders (Fig. S8). Thus, the key finding that soil bacterial communities exhibit strong biogeographic patterns is reproducible in data sets at local (longitudinal transect), regional (latitudinal transect), and global scales.

10.1128/mSystems.00540-20.8 FIG S8 Compositional turnover analysis at the continental scale. (a and b) Global scale occupancy frequency at two identity thresholds (100% and 97%). Taxa (ASVs) with lower than 0.05% relative abundance were retained in panel a and removed in panel b. (c) Normalized zeta decline at the continental scale across two identity thresholds (100% and 97%) and with either retention or removal of rare taxa (0.05%). (d) AIC values for exponential and power law model fits. Download FIG S8, TIF file, 1.5 MB. Copyright © 2020 Bay et al. 2020 Bay et al. This content is distributed under the terms of the Creative Commons Attribution 4.0 International license.

## DISCUSSION

In this study, we analyze patterns and drivers of soil microbial composition across multiple scales. Steps were taken to overcome common limitations in microbial biogeographical studies by leveraging innovations in sampling design, amplicon processing, and diversity metrics. We found the following. (i) Soil bacterial communities exhibit strong biogeographic patterns. (ii) Spatial turnover is rapid, as most taxa have low to moderate levels of occupancy. (iii) Community structure is influenced more by niche differentiation due to environmental variation rather than dispersal limitation. Our findings agree with previous literature that reported the uneven distribution of bacteria across communities and the strong influence of deterministic drivers (3, 12). However, we observed much stronger spatial turnover than reported in most, though not all, previous literature (13, 15, 28). This is reflected by the compatible findings of four independent analyses using the original high-resolution data set. Occupancy frequency distributions revealed most taxa were shared across less than 10% of samples (Fig. 1). Through zeta decline analysis, we detected a logarithmic decrease in the number of taxa shared as the number of sites increased (Fig. 3). In addition, we observed strong distance decay (Fig. 4a) and taxon-area relationships (Fig. 4c), with *z* values one to two orders of magnitude higher than most previous observations (13, 29, 34, 35, 37).

Multiple factors may explain why we observed high environmentally driven turnover. These potentially include the choices of sampling site, sampling scheme, sequence processing, and downstream analyses. It is notable that our desert sampling sites contained loessial soils that facilitate dispersal and the regional transect contained high environmental heterogeneity, which is known to be associated with increased bacterial turnover (31, 66, 69); however, this is unlikely to primarily account for most discrepancies with previous literature, given that rapid turnover was also observed in the local transect where physicochemical variation was lower and similar findings were also made in the global analysis. A more significant factor may be that our study adopted a hierarchical sampling design in order to quantify microbial variation across multiple spatial scales. In this regard, it is well-recognized that sampling design and sample size are critical determinants of taxa-area relationships (44, 74); this reflects that the detection of rare taxa largely determines species evenness and spatial structure, which in turn affects the exponent *z* (35). Methodological advances that improve the detection and inclusion of rare taxa are therefore predicted to align microbial *z* values more closely with those reported for animal and plant communities (44, 48). it is notable that other studies reporting high taxon-area exponents also used spatially explicit hierarchical designs (34, 35).

**FIG 5:**
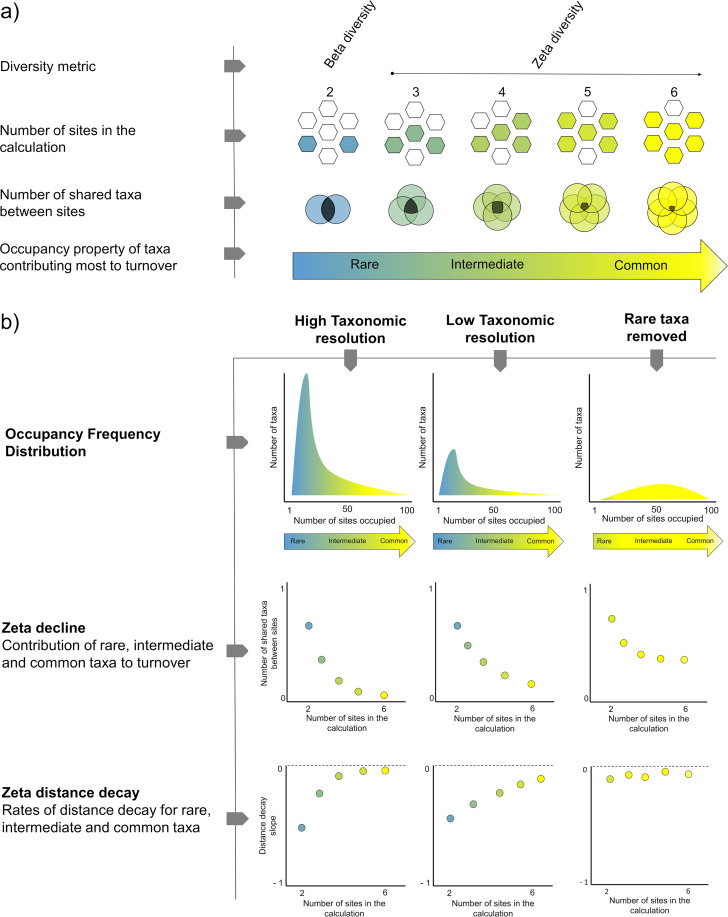
Summary of biogeographic patterns of soil microbial communities at different taxonomic resolutions. (a) Principle of how zeta diversity encompasses turnover of rare, intermediate, and common community members. (b) Comparison of patterns of occupancy frequency, zeta decline, and distance decay for rare, intermediate, and common community members. In addition, the figure demonstrates how the common approaches of clustering and filtering can bias biogeographic interpretations of microbial communities.

However, the biggest factor likely underlying these discrepancies is the treatment of sequencing data. A pervasive feature of 16S rRNA gene amplicon surveys is the clustering of similar sequences to remove potential “noise” and, less commonly, the filtering or undersampling of low-frequency sequences that constitute the rare biosphere. As summarized in Fig. 5, such processing greatly reduces and distorts the information in data sets, obscuring patterns in occupancy, turnover, and drivers. We avoided such downfalls by using a recently developed denoising algorithm to resolve sequence variants (59), while confirming through rarefaction curves that our sequencing efforts captured most rare taxa within and between samples. Through simulating sequence processing, we observed major differences in occupancy frequency, zeta diversity, distance decay, and taxon-area relationships upon filtering rare taxa and, to a lesser extent, clustering similar sequences (Fig. 5). It should be noted that these observations may appear to conflict with those of a recent study that reported clustering did not “change the rate of microbial taxonomic turnover” (28). However, this may be an issue of interpretation of distance decay curves. In common with this study (28), we also observed that the distance decay coefficient of bacteria and archaea remains similar between taxonomic resolutions, reflecting similar observations reported in fungal (75) and plant (76) communities. However, as the community similarity (*y* intercept) is lower at higher resolution, a higher proportion of taxa are lost overall in unclustered data sets compared to clustered data sets. Thus, it is reasonable to conclude that clustering masks microbial taxonomic turnover and broader biogeographic patterns.

This study also highlights the different patterns and drivers of community turnover between rare, common, and intermediate community members. As demonstrated by the occupancy frequency distribution, filtering sequences removes most rare species and retains most common ones. On the basis of the results of beta diversity analysis, we observed significant differences in the proportion of variation assigned to environmental, spatial, shared, or unexplained components at different taxonomic resolutions. This agrees with recent reports that environmental and spatial drivers differentially act on common and rare taxa (77, 78). Abundant generalists and rare specialists have been shown to differentially respond to environmental change, reflecting differences in niche breadth (11, 79, 80). Beyond these pairwise observations, we used zeta diversity to demonstrate that the turnover patterns reflect those typically observed in deterministically structured communities. Zeta decline consistently follows a power law, which indicates that communities are nonrandomly structured such as those with clear niche or range differentiation. However, upon lowering taxonomic resolution, these patterns degrade and increasingly resemble stochastic patterns such as seen in habitats with strong aeolian forces or aquatic flows (Fig. 5). These findings suggest that at lower taxonomic resolutions (49) or when rare taxa are removed (35), the community structure becomes more similar and thus predicted assembly processes switch from deterministic to stochastic. Through incorporating a multisite distance decay model, significant differences in the spatial structure of rare, intermediate, and common taxa could also be detected.

Looking forward, this work demonstrates how microbial biogeography can be advanced using readily implementable approaches. There is scope to use the methodological and theoretical innovations shown here to investigate these patterns across a broader range of environments and temporal scales. Detailed studies are needed to better capture the biotic and abiotic subsets of drivers responsible for changes in community turnover across all occupancy classes; this has been achieved in plant ecology (61, 81) but is currently lacking in microbial ecology. Likewise, it is critical to compare the patterns and drivers of community turnover in parallel for microorganisms and macroorganisms. Indeed, a key observation of our study is that the *z* exponent for the taxon-area relationships microbial communities falls within the interquartile range of higher animal and plant communities, suggesting that microorganisms and macroorganisms exhibit similarly strong spatial structure. However, given that these exponents are highly sensitive to factors such as sampling design, sample size, and taxonomic resolution (44, 74), a rigorous comparison of turnover between domains requires side-by-side sampling. Finally, emerging advances in long-read and full-length 16S rRNA gene sequencing may enable resolution of biogeographic patterns of microorganisms at both the species and strain levels (82, 83).

## MATERIALS AND METHODS

### Soil survey design.

Topsoil samples were collected along perpendicular latitudinal and longitudinal transects in the Judea Hills and Negev Desert regions, Israel. The latitudinal transect, which was designed to capture a high level of environmental heterogeneity, extended for 160 km in a north/south direction along a steep aridity gradient. This transect traversed four climatic zones that were differentiated by mean annual precipitation patterns: subhumid shrubland (300 to 400 mm/year), semiarid grassland (∼200 to 250 mm/year), arid desert (∼50 to 90 mm/year), and hyperarid desert (<20 mm/year). The longitudinal transect, sampled within the arid zone across a relative homogenous climate, extended perpendicular to the latitudinal transect for 20 km in an east/west direction.

A hierarchical sampling scheme was used to capture biogeographic patterns across multiple spatial scales and provide sufficient spatial resolution to cover the majority of distance classes between sites (see Fig. S1c in the supplemental material). Three spatial hierarchies were within each climatic zone: (i) site level (two representative sites of ∼1,000 m^2^), (ii) plot level (three representative plots of ∼100 m^2^), and (iii) sample level (random triplicates of ∼100 cm^2^) (Fig. S1b). Site selection was based on four criteria: (i) soil type (wind-deposited loessic soils in the subhumid, semiarid, and arid zones and gypsic soils in the hyperarid zone), (ii) presence of soil crust to indicate no recent disturbance, (iii) vegetation-free soil to minimize a vegetation effect, and (iv) a buffer of 100 m from roads, slopes, and seasonal runoff water channels. No statistical methods were used to predetermine sample size.

### Soil sampling and analysis.

In total, 99 topsoil samples were collected across both transects over a 10-day period in May 2017. Prior to sampling, GPS coordinates and site metadata were recorded. Soil samples of approximately 50 g were collected in triplicate, using sterile techniques, by removing the soil crust (0- to 2-cm depth) and then sampling the underlying topsoil (2- to 10-cm depth). Samples were placed into individual 50-ml screw top falcon tubes and stored at 4°C until downstream analysis.

Within 24 h of sampling, all soils were homogenized by sieving (500 μm) and soil water content (as a percentage) was measured gravimetrically in duplicate. All samples were then shipped to quarantine approved facilities at the School of Biological Sciences, Monash University. For soil chemistry analysis, samples were pooled to form one representative sample per plot and sent to the Environmental Analysis Laboratory, Southern Cross University. In total, 21 separate soil chemical parameters were selected for analysis, based on commonly reported drivers of soil microbial communities globally and those reported by previous studies of the Judea Hills and Negev Desert (66, 84). These parameters included the following: soil acidity (pH), electrical conductivity (EC), effective cation exchange capacity (ECEC), total organic carbon, total nitrogen, sodium (Na), sulfur (S), phosphate (P), potassium (K), nitrate (NO_3_^−^), and ammonium (NH_4_^+^), as well as bioavailable minerals, including manganese (Mn), copper (Cu), zinc (Zn), boron (B), aluminum (Al), iron (Fe), and silicon (Si). Each chemical parameter was calculated following the methods of Rayment and Lyons (85). Aridity data for each site were obtained from a global geospatial data set (86) mapping the aridity index (MAP/PET, where MAP stands for mean annual precipitation and PET stands for potential evapotranspiration) at a resolution of 90 arcseconds (approximately 1 km at the equator) using a climatic time series from 1950 to 2000 (86). This data set includes samples from six continents, Africa, Europe, Asia, Australia, North America, and South America.

### Community DNA extraction and sequencing.

For all samples, total community DNA was extracted from 0.25 g of soil using the modified Griffith’s protocol (87). We confirmed the DNA yield, purity, and integrity for each extraction using a Qubit fluorometer, Nanodrop 1000 spectrophotometer, and agarose gel electrophoresis. For each sample (88), the hypervariable V4 region of the 16S rRNA gene was amplified using the universal Earth Microbiome Project primer pairs F515 and R806 (89). The amplicons were subjected to Illumina paired-end sequencing at the Australian Centre for Ecogenomics, University of Queensland. Twelve samples were also subjected to shotgun metagenomics sequencing (SH.1.A3, SH.1.C2, SH.2.C3, SA.2.B3, SA.1.C3, SA.1.B1, AR.2.A3, AR.2.A1, AR.1.C2, HA.2.C2, HA.1.B1, and HA.1.C2). DNA was extracted from 0.25 g of soil using the MoBio PowerSoil isolation kit according to the manufacturer’s instructions. Metagenomic shotgun libraries were prepared for the 12 samples using the Nextera XT DNA sample preparation kit (Illumina Inc., San Diego, CA, USA). Sequencing was performed on an Illumina NextSeq500 platform with 2 × 150 bp high-output run chemistry. For analysis of the previously published global data set (7), the raw 16S rRNA gene amplicon sequences were downloaded from Figshare (https://figshare.com/s/82a2d3f5d38ace925492).

### Amplicon-based community profiling.

Raw sequences from the Israel and global data sets were processed on the QIIME 2 platform (90) using the deblur pipeline (59) to resolve exact amplicon sequence variants (ASVs). In contrast to operational taxonomic unit (OTU)-based approaches that cluster sequences to a fixed identity threshold (usually 97%), deblur controls error rates (typically 0.1% per nucleotide) to resolve single-nucleotide differences over the sequenced gene region (59).

Paired-end raw reads were demultiplexed, and adapter sequences were trimmed, yielding 3,989,659 reads across all samples. Forward and reverse reads were joined using the q2-vsearch plugin (91). A quality filtering step was applied using a sliding window of four bases with an average base call accuracy of 99% (Phred score of 20). Low-quality reads were removed, and sequences were truncated at 250 bp before denoising using deblur (59). For downstream analysis, three samples with low read counts (<1,000 reads) were excluded (SH.1.B2, AR.1.B1, and AR.1.B2). An additional 414 ASV singletons were detected and removed. The final data set contained 96 samples and 11,335 ASVs (see Data Set S1, tab 1, in the supplemental material). In order to compare biogeographic patterns across different taxonomic resolutions, a second data set was created by clustering the ASVs at a 97% identity threshold using open reference OTU picking via q2-vsearch (91) (Data Set S1, tab 2). The third and fourth data sets were created by removing reads with lower than 0.05% relative abundance from the 100% and 97% identity threshold data sets using the Phyloseq filter_taxa function.

Taxonomic assignment was performed as per a previously described approach (https://osf.io/25djp/wiki/home/). Briefly, all reference reads that matched the 515F/806R primer pair were extracted from the Genome Taxonomy Database (GTDB) (92) and used to train a naive Bayes classifier (93) by using the fit-classifier-naive-bayes function with default parameters. The classifier was then used to assign the taxonomy to the ASV feature table. Representative sequences were aligned using the multiple sequence alignment program MAFFT (94), and a phylogenetic tree was constructed using the fast-tree method in QIIME 2.

### Metagenome-based community profiling.

The 16S rRNA gene amplicon sequence is commonly used as a marker to profile microbial communities. However, a major limitation of this approach lies in the intragenomic and intergenomic variation in copy number of the 16S rRNA gene (73, 82). We conducted a comparative metagenomic analysis on a subset of 12 samples (biological triplicate from within each climatic zone) along the latitudinal transect using a single-copy ribosomal marker in order to test whether our observations of community turnover by 16S rRNA gene amplicon sequencing were affected by this variation. A total of 318,420,199 reads were obtained from metagenomic sequencing across the 12 samples. In contrast, the read counts for the negative controls were 6,547 (extraction control) and 1,360 (library preparation control). Raw sequence reads in each sample were stripped of adapter and barcode sequences, then contaminating PhiX sequences were identified and removed using the BBDuk function of BBTools v. 36.92 (https://sourceforge.net/projects/bbmap/) with a kmer size of 31 and hamming distance of 1. We then used SingleM (95), which uses hidden Markov model (HMM) searches of single-copy ribosomal markers, to generate *de novo* OTUs. In total, 28 HMM searches were performed against 14 single ribosomal single-copy marker genes. GraftM was used for taxonomic annotation of OTUs by searching sequences using hmmsearch (HMMER) (96). For downstream analysis, the single-copy marker gene *rplP* was used for comparison, encoding ribosomal protein L16 L10e. This marker was previously identified as a robust means of distinguishing between both closely and distantly related genomes (97). Sequences were then clustered *de novo* into OTUs using a sequence identity threshold of 97%. Taxonomic assignment was carried out using the GTDB taxonomy.

Due to large differences in sequencing depth, the amplicon and metagenomic sequences analyzed were both rarefied at 300, which was the minimum number of sequences observed for *rplP*. Rarefied data sets were used only in the supplemental analysis shown in Fig. S7, whereas the rest of the study used unrarefied data sets.

### Richness analysis.

Statistical analysis and visualizations were performed in R version 3.4.4 (2018-03-15) using the packages ggplot2 (98), phyloseq (99), vegan (100), and zetadiv (55). Occupancy frequency distributions (64) were used to visualize the distributions of the numbers of taxa occupying different numbers of areas and examine the distributional shifts at lower identity thresholds and after filtering rare taxa. Taxa accumulation curves were used to compare alpha diversity properties between sites and confirm adequate sampling of the microbial community. A sample-based rarefaction method was used to find the expected curve, namely, the Mao Tau estimate, and a moment-based standard deviation was estimated from the extrapolated number of ASVs surveyed (gamma diversity) using the “exact” method of the *specaccum* function [Vegan | R] (100). Observed richness and estimated richness (Chao1 and abundance coverage estimate [ACE] methods) were calculated using the *estimate_richness* function [Phyloseq | R] (99). To test for significant differences in the mean observed and estimated richness at the site level, an analysis of variance (ANOVA) with a Shapiro-Wilk test to confirm normality was used.

### Turnover analysis.

The multisite diversity metric zeta diversity (ζ) was used [Zetadiv | R] (55) to examine incidence-based turnover in community composition (Fig. S5). Pairwise metrics of incidence-based turnover (e.g., Jaccard, Simpson index) are biased toward detecting turnover that is driven predominantly by the loss and addition of taxa from the rare biosphere, as by definition rare taxa are not shared by many sites. Zeta diversity overcomes this limitation by enabling discrimination between turnover of rare, intermediate, and common taxa. With increasing orders of zeta, the average number of taxa shared between sites declines and the contribution of increasingly more common taxa to the value of zeta diversity increases. Variation in the rate and form of zeta decline provides information on community structure and inference of the processes driving community assembly. If the zeta decline follows an exponential form (the ratio between ζ_i_ and ζi_-1_ is constant), there is a similar probability of finding a common or rare taxon with the addition of a site, suggesting that turnover is predominantly stochastic or dispersal limited. However, if zeta decline follows a power law form (the ratio between ζ_i_ and ζi_-1_ increases at higher orders), then the chance of detecting a common taxon is greater than detecting a rare one with increasing orders, demonstrating structure in the community and suggesting that turnover is driven primarily by deterministic processes such as selection due to soil or climate (55).

Zeta decline using Monte Carlo sampling was calculated via the *zeta.decline.mc* function [Zetadiv | R] (55). Zeta diversity was calculated on nonweighted presence-absence data for ζ orders ζ_1_ to ζ_6_; this captured the extent at which the community was structured across each transect, as ζ values within each data set approached zero. To account for differences in richness between sites, all ζ*_i_* values were normalized by using a Jaccard normalization with subsampling set to 1,000 permutations for each analysis. Power-law and exponential models were fitted to ζ*_i_* decline curves and Akaike information criterion (AIC) were used to estimate the likelihood of either exponential or power law model describing the relationship between ζ diversity and order *i*.

### Biogeographic analysis.

We calculated the distance decay of similarity across both transects to quantify the number of shared ASVs over geographic distance and to explore turnover within the context of geographic distance. Pairwise distance decay was calculated using normalized ζ_2,_ with subsampling set to 1,000 using the function *zeta.ddecay* [Zetadiv | R] (55). To quantify the contribution of rare and common ASVs to turnover, distance decay was calculated for orders ζ_1_ to ζ_6_ by using the mean distances between pairs of *n* sites via the *zeta.decays* function [Zetadiv | R] (55). Spatially explicit taxa-area relationships (74) were calculated by estimating richness as a function of the sample, plot, and site level spatial hierarchies using the *specnumber* function [Vegan | R] (100). The taxa-area curve was fitted using the Arrhenius model with the expression *kA^z^*, where *k* is the average number of taxa, *A* is the area (spatial hierarchy), and *z* is the steepness of the curve. For comparison, turnover rates from this study were compared against a total of 655 data sets, including bacteria and higher eukaryotes (101).

### Community structure analysis.

Principal-coordinate analyses (PCoA) were used on both weighted and unweighted distance matrices. Read counts were normalized to relative abundance, and a square root transformation was applied prior to calculating distances between samples using Bray-Curtis. For nonweighted analysis, read counts were transformed to incidence (presence-absence) and distances were calculated using the Jaccard index. A multivariate model-based framework was used to test for significant differences in community structure among spatial hierarchies and identify the subset of environmental drivers that best explain spatial patterns in community structure [MVAbund | R] (102). Microbial abundance and incidence data typically show a mean-variance relationship, which standard approaches such as permutational multivariate analysis of variance (PERMANOVA), analysis of similarity (ANOSIM), and redundancy analysis (RDA) fail to account for. Instead they rely on pairwise distance matrices which convert multivariate data sets to univariate ones, which has been shown to reduce statistical power. MVAbund solves this problem for nonnormal data by fitting a single generalized linear model (GLM) to each ASV separately and performing resampling of *P* values to determine significance of a shared predictor variable.

In this study, ASV incidence data were modeled using generalized linear models. Mean variance relationships of the data were confirmed by visually inspecting scatterplots showing mean variance as a function of ASV incidence. Model assumptions were validated by inspecting Dunn-Smyth residuals as a function of each predictor variable, and significance was established using a likelihood ratio test (LRT) with PIT-trap bootstrapping (103). To obtain the subset of predictor variables which best explain a multivariate response, significant predictor variables were passed through a forward selection in a multivariate linear model using the top 10 independent variables with the highest average *R*^2^. A variation partitioning analysis was performed to disentangle the autocorrelation between environmental and geographic distance and partition variation in community structure into its spatial and environmental components. Multisite generalized dissimilarity modeling (MS-GDM) was used to identify the importance of correlates of turnover by regressing ζ_2_ against the subset of identified predictor variables at each taxonomic resolution using *zeta.msgdm* function [Zetadiv | R] (55). Subsequently, a variation partitioning analysis was performed using the *zeta.varpart* function [ZetadivR] (55), which partitioned the variation into (i) variation explained by distance alone, (ii) variation explained by either distance or environment, (iii) variation explained by environment alone, and (iv) unexplained variation.

### Data availability.

The amplicon and shotgun sequencing data sets generated for this study have been deposited in the NCBI Sequence Read Archive under the BioProject accession no. PRJNA642232.
